# Editorial: Functional screening for cancer drug discovery: from experimental approaches to data integration

**DOI:** 10.3389/fgene.2023.1201454

**Published:** 2023-07-06

**Authors:** Kecheng Zhou, Wenyu Wang, Jing Tang

**Affiliations:** ^1^ School of Life Sciences, Anhui Medical University, Hefei, China; ^2^ Research Program in Systems Oncology, Faculty of Medicine, University of Helsinki, Helsinki, Finland

**Keywords:** functional screening, cancer drug discovery, experimental approaches, data integration, editorial

Cancer is still one of the leading causes of death worldwide, despite that tremendous resources are being invested in drug discovery (Siegel et al., 2022). Recent developments in uncovering the molecular biology of oncogenesis and tumor development (Zhou et al., 2018; Liu M. et al., 2022; Yan et al., 2023), and the studies on cancer polypharmacology (Pushpakom et al., 2019; Cohen et al., 2021) have challenged researchers to come up with new strategies for integrating ever-growing data at the molecular levels.

The majority of cancer medications consist of small molecule inhibitors. These inhibitors are engineered to enhance their efficacy by binding to specific cellular protein targets, which in turn initiate a series of downstream changes in cancer signaling and metabolic pathways (Yang et al., 2019; Goel et al., 2020). High-throughput phenotype-based drug screening has proven successful in identifying compounds that demonstrate promising cytotoxic effects (Letai et al., 2022). However, it is essential to further investigate the processes occurring between drug administration and the ultimate phenotypic response. Additionally, determining the genetic factors that influence variations in drug sensitivity or resistance is crucial for advancing cancer treatment strategies (Kuusanmäki et al., 2020).

Depending on the study’s focus, high-throughput genetic perturbation experiments can yield results related to either 1) proliferation-associated phenotypes, or 2) intermediate phenotypes, such as transcriptomic alterations. Numerous well-established data portals, including The Cancer Genome Atlas (TCGA), DepMap Portal (Gonçalves et al., 2020), and The cBioPortal for Cancer Genomics (Cerami et al., 2012), have curated and standardized experimental data from tissues and cell lines, facilitating an easy access for subsequent systems medicine approaches (Wang et al., 2019). Moreover, cutting-edge studies are increasingly generating new data types, such as those derived from 3D organoids (Weeber et al., 2017; Hahn et al., 2021) and *in vivo* patient-derived xenograft (PDX) samples (Gao et al., 2015; Zanella et al., 2022). These innovative techniques hold the potential to bring researchers closer to a more biologically relevant foundation, moving beyond flat biological platforms. To fully utilize data from these advanced methodologies in identifying genetic signatures and molecular mechanisms for cancer drug discovery, versatile and robust data integration and curation efforts are developped (Zheng et al., 2021; Tanoli et al., 2022). These tools are expected to pave the way for computational and artificial intelligence research, propelling the field towards clinical translation (Ma et al., 2021; Douglass et al., 2022). The selected acticles in this Research Topic will focus on both experimental approaches and data integration, aiming at facilitating the drug and target discovery in cancer.

The goal of the Research Topic “*Functional screening for cancer drug discovery: from experimental approaches to data integration*” is to highlight the recent advances in high-throughput functional genetic approaches, especially how results from such new technologies can be applied for future studies in cancer drug mechanisms of action. To achieve this goal, we carefully reviewed every submitted manuscript and screened for highly qualified reviewers. Eventually, we accepted and published nine articles including eight “Original Research” articles, and one systematical “Review” article on the mechanism and clinical trials of hepatocellular carcinoma immunotherapy (Huang et al., 2021).

The research articles presented in this Research Topic encompass efforts in cancer drug discovery from both experimental and computational perspectives. Jiang et al. identified the crucial role of RNA polymerase II subunit A (POLR2A) in the onset and progression of gastric cancer (GC). Ren et al. developed a necroptosis-related prognostic signature to reveal immune infiltration, which could predict drug sensitivity and inform personalized drug therapy for hepatocellular carcinoma (HCC) patients. Wang et al. reported a novel hub gene signature closely associated with ferroptosis, serving as a potentially effective biomarker for predicting the prognosis of HCC patients.

Liu et al. conducted a comprehensive analysis to elucidate the roles of cuproptosis-associated genes in tumor biology and cancer drug sensitivity across various cancers. Chen et al. discovered a novel diagnostic four-gene signature for hepatocellular carcinoma based on an artificial neural network, with applications in drug screening. Li et al. explored the functional effects of FDX1 in tumors, and further validated the inhibitory effect of FDX1 in copper-induced cell death, confirming FDX1’s role as a cuproptosis biomarker. Ruan et al. leveraged pan-cancer analysis to identify DDX56 as a prognostic biomarker associated with immune infiltration and drug sensitivity. Zhang et al. uncovered an LncRNA signature related to cuproptosis, serving as a novel biomarker of prognosis in immunotherapy and drug screening for clear cell renal cell carcinoma.

We believe that the articles featured in this Research Topic can offer valuable insights into the application of functional screening methods for cancer drug discovery. The findings presented in these studies are anticipated to enhance our understanding of the molecular mechanisms governing cancer progression and, ultimately, we hope they will positively impact drug and target discovery in the near future.
